# Supplementary material for: Olfactory channels associated with the Drosophila maxillary palp mediate short- and long-range attraction
Source: eLife. 2016 May 23;5:e14925. doi: 10.7554/eLife.14925 (PMC4927298; doi:10.7554/eLife.14925)
Supplement: Supplementary file 1. — DOI: http://dx.doi.org/10.7554/eLife.14925.029 [file elife-14925-supp1.docx]

>D.suzOr33c (cDNA, full length)

ATGGTCATCATCGATAGTGTTCATTTTTATCGTCCGTTCTGGCTCTGCATGCGAGTGCTAGTACCGACATTCTTCAAGGAGTCAAGGAGTCTTCAAGCAGGCCCCATCCAGCTTTATGTGGCGGCGCTACATATTCTGGTCACCTTGTGGTTCCCACTGCACCTTCTGCTGCATCTTCTGTTGCATCCTTCTGCGGCTGAACTAGTGAAGAACCTATCCATGTCCATAACCTGTCTTGCCTGCAGTCTCAAGCATGTGGCCCATCTGTATCACTTGCCTGAGATTGTGGAGATCGAATCTCTGATCGGACAGCTGGATACGTTCGTATCGAGCGAACAGGAGCATCGATACTACCAGGATCACGTGCACTGTCATGCGAAGCGCTTTACGCGATGTCTCTACATAAGTTTCGGTATGGTGTACGTGTTGTTCCTATTAGGTATTTTCGTTCAGATCATTAACGGAAACTGGGAGCTCATATACCCCGCGTATTTCCCTTTTGACTTGGAGAGGAATCAATGGATGGCTGCATCGGCGCTGGGCTATCAGGTTATCAGCATGCTTGTCGAAGGCTTTCAGGGTCTGGGAAACGACACCTACACCCCGTTGACCTTGTGTCTTCTAAGCGGACACCTCCATTTGTGGTCCATTCGGATGGCTCAACTCGGATTCCTCGAGGACGAAACTGCCAGTAATCATCAGCGGTTGCTGGAATACATCGAGCAGCATAAGCTCTTGGTCAGATTCCACAGCTTGGTGGCTCAGACCGTCAGCCCGGTGCAGCTGTTCCAGTTGGGCGGCTGTGGCGCCACCCTGTGCATCACCGTGACCTACGTGCTCTTCTTTGTGGGCGACACCATCTCGCTGGTCTACTACTTGGTCTTCTTCGGGGTGGTCTGCATGCAGCTCTTCCCCAGCTGCTACTTTGCCAGCGAGGTGGCCGAGGAGGTGGAGAGCCTGCCGTATGCGATCTTCTCCAGCAGGTGGTACGATCAGTCCCGGGAACATCGATTCGATCTGCTCGTCTTCACCCAACTGACCCTGGGGTCCAGGGGACGGGTCATAAAGGCGGGCGGGCTCATCGAGCTGAATTTAAATGCCTTTTTCGCCACTCTGAAGATGGCCTACTCCCTGTTCGCCGTCGTGGTGCGGGTGAAAGGTATATAG

>D.suzOr42a (cDNA, full length)

ATGGAGCTGCAAAGAATCATTCCGGCCCTGTTTACGCAATCGGAGGACTCCCCAGTTCGCTCGCGGGACGCGACCCTGTACCTGCTGCGCTGCATCTTCCTGATGGGCGTACGCAAGCCCCCTGCAAAATTCTTCGTGGCCTACGTCCTTTGGTCCATTGCCCTGAATTTCTGTTCCACATTTTACCAACCCATCGGATTTCTCACGGGCTACATCAGCCATTTGTCGGAGTTCTCGCCGGGAGAGTTTCTTACTTCGCTCCAGGTGGCGTTTAACGCGTGGTCCTGCTCCACCAAGGTGTTGATTGTGTGGGCACTGGTCAAACGCTTTGACGAGGCTAATGCCATCCTCGACGAGATGGATAAACGTCTCACAGAGCCCGGCGAGCGACTACAGGTTCACCGCGCTGTCTCCCTTAGCAACCGGATATTTTTTTTTTTCATGGCAGTGTACATGGTTTACGCCACGAATACGTTCCTTTCAGCTATCTTTATTGGAAGGCCTCCTTACCAAAATTACTATCCGTTCCTGGACTGGCGATCCAGCAGATTGCACCTCGCCCTACAGGCCGGCCTGGAGTACTTCGCCATGGCTGGTGCCTGTTTTCAAGATGTTTGTGTGGATTGCTACCCCGTTAACTTCGTCCTCGTGCTTCGGGCACATATGTCTATCTTTGCGGAGCGTCTCCGGCGTCTTGGCACAGATCCTTCGGAGAGCACGGAGCAGCGATTCGAGCGATTAGTGGAATGCATACATGACCACAAGGCCATACTGCGATTTGTCAACTGTCTGCGCCCTGTTATCTCTGGAACCATATTTGTGCAGTTCCTGGTAGTGGGATTGGTGCTGGGCTTCACTCTAATCAACATTGTTTTGTTTGCTAATTTGGGATCGGCTATCGCAGCGCTGTCCTTCATGGCTGCTGTGCTTCTGGAGACGACGCCCTTTTGTATTCTGTGCAACTATCTTACAGAAGACTGCTATATACTGTCTGATGCCTTGTTTCATTCCAATTGGATCGATGGGCAGAGGCGCTACAGAAGTACTGTCATGTACTTCCTACAGAAACTACAACAGCCGATAACCTTTATGGCTATGGACATATTTCCGATATCGGTGGGAACTAACATCAGTGTCACCAAGTTTTCGTTCTCCGTCTTTACTCTCGTAAAGCAAATGAATATCGCTGAGAAATTAGCCAAATCTGATGAAGACGATTGA

>D.suzOr46a (cDNA, full length)

ATGAGCAACAGAGTGGAAATCTTTTACAAGGGGCAGAAGGCCCTCCTTAACGTATTCTCGCTTTGGCCCCAAAACGAACGCCATTGGAGAATCTTTCATCAGGTTAACCACGTACATGTGATGGGATTTTGGGTGCTGCTCTTTGATCTCCTGCTCGTGATGCATGTGGTGGGCAACCTGAGCTCGATGTCCGAGGTTGTAAGGGCCATCTTTGTTCTGGCCACCAGTGCGGGACACACGACCAAGCTGCTGAGCATCAAGGCCAATAATGTGGAACTGGAGGAGCTATTCAAGAGGTTGGACGAGGATGATTTTCGACCAAGGGGCGTTGAAGAGGAGTTGATCCTGGCAGCTGCCTGTGAAAGGAGTAGGAAGCTCAGGGATTTTTATGGCACTCTGTCGGTGGCCGCCTTGTCCATGATCCTTATACCCCAATTTGTGGTGGACTGGTCCCAACTACCACTGGGCACATATAATCCGTTTGCCGACCATCCTGGTTCACCCGGTTACTGGCTCCTCTATTGCTATCAATGCCTGGCCCTGACCGTCTCCTGTGTCACCAATATAGGCTTCGATTCCCTCAGCTCCTCGCTGTTCATCTTCATCAAGCGCCAGCTGGATCTTCTGGCGGTGCGACTGGATAAGATGGGCCGGTGGAACAGTGCCGGTGGCTCCGTGGAGCAGCAACTGAAGCAGAATATCCGGTACCACATGGCCATTGTGGAGTTGACAGCAACTGTGGAGCGCCTTCTTTGCAAGCCGATTTCGATGCAGATCTTTTGCTCCGTTTTGGTTCTCACTGCCAATTTCTATGCTATAGCTCTGTTGTCCGACGAGAAGCTGGCGCTATTTAAATATATTACCTATCAGGCGTGCATGCTGAGTCAGATTTTTATATTGTGCTACTATGCCGGAGAGGTAACCCAGCGAAGTTTGGACCTGCCCCATGAGCTTTACAAAACATCTTGGGTGGACTGGAACAAGGATTCACGAAGGATTGTGCTGCTTTTTATGCAACGCCTTCATTCGACTCTGAGAATAAGGACCTTAAACCCCAGCCTGGGTTTCGATCTAATGCTTTTTAGCTCGATTGTAAACTGCTCTTACAGCTATTTCGCCTTGCTAAAGCGGGTCAACAGTTAG

>D.suzOr59c (cDNA, full length)

ATGAAGAAGCCGCTCTTTGAACGTCTGCGAACTGCACCACTTGATAACCATAGCATCAGTTCCTTGGTTGCCAGTGATTACTTTTATCGCATTGCATTCTTCATGGGTTGGAATCCTCCCACGGAAGGTTTCTTCAGACGGATATACTTCTTATTGACTTTTACCACGATGTTGCTGGGGATTGTATGTCTACCGCTGGGTCTAACGCTCACCTATGTAAAGCACTTTAGTAGGTTTACGCCGACTGAGTTTCTGAGCTCCCTGCCAGTGGACGTCAACTGTATTGGAAATTTGATCAAGACTTGTGTGACCTTCACGTCGATGTGGCGATTTCGCTTGATGAATGAACTGATTTCCCCTTTGGATAAGAGATGTGTTAGCCCATCGCAGCGTCAGATTCTCCATAAATCGGTGGCTCGGGTTAATCTAATTGTGATTGTGTTTCTGTCTACTTACTTGGTTTTCTGTTTCCTGAACCTTTTTACATCGGTTCTTTCGGGCACGGCACCTTGGCAATTGTACAATCCTCTTGTGGACTGGCGGAGTGGCTATTGGCAACTCTGGATTGCCTCGCTTATGGAATACGTTGTGGTCTCTATAGGCACTTTGCAGGAATTGATTTCGGACGCCTATCCCATTGTCTTCATTTCCTTGTTCCGATGTCATCTGGCTATTCTGAGGGATCGCATAGAGAATCTGCGACAGAATCCGGAGCTCAGTGAGAAGGAGAACTACGAACAGTTGGTGGCCTGCATCCAGGATCATCGCACCATCATTCAATGTAGCGAAATCATTCGTCCCATCCTGTCCGTCACCATCTTTGCACAATTCATGCTGGTGGGCATCGACTTGGGATTGGCTGCCATAAGCATCCTGCTATTTGAGAATACCATCTGGACGGTCGTGGCGAGTGTCTCATTTATCATGGCCATCTGCATGGAATCCTTTCCTTGCTGCATGCTATGCGAACATCTCATCGAGGATTGTGTCCAAGTCAGCGATGCATTGTTCCACTCCAATTGGTTAACTGCAGATAGGCGTTATAAGTCGGCGCTTCTGTATTTTATGCACAGGGTTCAGAAACCCATTCAATTTACAGCTGGAGCGATATTTCCCATTTCGGTGCAGAGTAATATTGCAGTGGCCAAATTCGCCTTTACAATTATCACAATCGTCAAACAAATGAATCTCGGAGAGAAGTTTCTAAATAACAGGGCAAATGCAGGGGAAGTAGAGCCCTAA

>D.suzOr71a (cDNA, full length)

ATGGATTACGACCGAATTCGACCAGTGCGACACTTGACAGGAATTCTTGAATGGTGGCGTCTTTGGCCAAAGAGAGGATCGATTTCCAGACCGGATTGGACCAATTGGCGAGGATATCTTCTGCACATTCCCTTTACCTTGCTCTTTATGGTCCTTTTGTGGGTGGAGGCCATGATGAGCAGGGATATAGAACATACAGCCGATGTGCTTTTAATTTGCTTGACCACAACTGCTTTGGGGGCTAAAATGCTGAATAACTGGAAATATGCCCATGTGGCCCAAAAGATTTTAAGCGAATGGAGCACCTCGGATCTTTTTGAGTTAAGAAGCAAACAGGAAGTGGAAATGTGGAAATTCGAGCATCGAAGATACAGGCGCGTGTTCATTTTCTATGGTTTATGTAGTGGTGGTGTTATTCCTTTAATTGTGATCCAACCTCTGTTTGATATTCCCAATCGGTTGCCCTTTTGGATGTGGACACCCTTCAACTGGCATCAGCCGAATCTGTTTTGGTATCCATTTATTTATGAGGCCATAACCATTCCGATTGTCTGCATTTGCAATATTACCATGGATGGTATTAATTGGTATATGATGTTGCATCTATCGCTCTGTTTGCGAATGTTGGGTCAACGATTGAGTAGCCTTCGGCATGATGATAAAAAACTAAAGGAGAAGTTCCTGGAATTGGTGCATGTGCATCATCGACTTAAACTACAGTCTTTGGACATTGAGAACTTTATTTCGAAGAGTACTTTCACCCAAATTCTGGTCAGTTCCTTGATAATTTGCTTCACCATATACAGTATGCAGATGAGACCCGTCACCCAGGATCTGCCGGGATTTGCTGCCATGATCCAGTATTTATTAGCTATGATCATGCAAATCATGCTGCCCAGCATTTATGGTAATGCCGTCATCGATTCTGCACATATGTTGACCGATTGTATGTATAATTCTGATTGGCCGGATATGAATCCCCGAATCCGTCGACTCATCCTAATGTTTATGGTCTACTTAAATCGTCCCATGGCTTTAAAAGCCGGTGGCTTTTTTCATGTTGGCTTGCCTCTTTTTACCAAGACCATGAATCAAGCCTATAGCCTGCTAGCCTTGCTGCTCAACATGAACCAATAG

>D.suzOr85d (cDNA, partial)

ATTGGAATGCTGGCCTACGATCACAAGGATAGTGACAAAAAGAAGGAGCTCCTGCTCCACTGGATGTTTATTGGGCAGATGGTCAACCTGAATGCAGTGCTCATTTCGGAACTGATCTACGTATTCCTGGCGATCAGCCAGGGAAGCAACTTCCTGGAGGCCACCATGAATCTGTCTTTCATTGGTTTTGTGATTGTTGGGGATCTAAAAATCTGGCACATTTGGCGTCAGCGGAAGAGACTCACCCAAGTGGTAAACGAATTGGAGAACTTACATCCAGAACGCTTGGATCAGCAGGAGCCATATAATATCGAGTATCACGTAAATGGCTATAGCCGTTATAGCAAATTTTACTTTGGCATGCACTTGGTGCTGATCTGGACCTACAACCTCTACTGGGCGGTCTACTATCTGGTGTGTGATTTCTGGCTGGGAGTTCGTAAATTTGAGAGGATGCTGCCCTACTACTGCTGGGTGCCCTGGGATTGGAGTACCGGATTTAGCTATTACCTGATGTATATCTCGCAGAACTTGGCTGGACAGGCCTGTCTCTCGGGTCAGCTGGCAGCGGATATGTTGATGTGCGCCTTGGTCACTCTGGTTGTGATGCACTTCATCCGCCTTTCCGGGCACATCCAGGGGCATGTGACGGGGATTCTATCGCCACAGCAGGACTTGGAGTTCATCCAAGCGGTGGTGGCCTATCATCAGAGGTTATTGCTTCTCTGCCAGGACATCAACGAGATATTCGGGGTATCCCTGCTGTGCAACTTTGTGTCCTCATCCTTCATCATCTGCTTCGTGGGATTCCAGATGACCATCGGTGGCAAGATCGACAACCTGGTAATGCTCGTCCTGTTTCTCTTTTGCGCTATGGTGCAGGTCTTTATGATTGCCACACACGCCCAACGGCTCATAGATGCGAGTGAGCAGATTGGCCAAGCAGTCTACAACCACGACTGGTTCCATGCAGATTTACGTTACCGTAAGATGCTAGTTCTGATCGTTAAGAGGGCCCAACAACCCAGTCGCCTAAAGGCCACAATATTCCTCAATGTCTCACTGGTCACCGTATCGGATCTGCTACAACTATCCTACAAATTCTTCGCCCT

> DsuzOr85e (cDNA, partial)

ATGGCCAGTCTTCAGTTCCACGGCAACTTCGATGCGGACATCAGGTATGATATTAGTCTGGATCCGGCGAGGGAATCGAATCTTTTCCGCCTGCTGATGAGATTGCAGTTGGCGATGGGTATGAAACCATCTCCTCGGGTGCCCAAATGGTGGCCAAAGTGGGTGCAAATGGTGGGCAAACTTCTGGCCAAGGTCTACTGCTCCATGGTTATTTTTACCTCGCTGCATCTGGGAGTCCTGTTTACGAAAACCACATTGGATGTCCTGCCAACGGGTGAACTGCAGGCCATTACCGATGCCCTCACCATGACCATAATATACTTTTTCACCGGCTATGGTACCATCTACTGGTGCCTCCGCTCCCGACGCCTCCTGGCCTACATGGAGCACATCAACCGCGAGTATCGCCACCATTCGCTGGCCGGGGTGACCTTTGTGAGCAGCCATGCGGCCTTCCGGAAGTCCTGGAACTTCACGGTCGTGTGGATAATGGCCTGCCTGCTGGGCGTGATCTCCTGGGGCGTCTCGCCCCTGATGCTGGGCATCCGGATGCTGCCCCTGCAGTGCTGGTATCCCTTCGACGCCCTGGCCCCCGTCACATATTCGGCGGTGTATGCCACCCAGCTGTTCGGTCAGGTCCTCGTGGGCGTGACCTTTGGCTTCGGGGGATCGCTTTTCGTCACCCTCAGCCTGCTGCTTCTGGCCCAGTTCGATGTGCTCTACTGCAGCCTGAAGAACCTGGACGCCCATGCCAAGCTGCTGGCCGGGGAGTCCGTTAATGGTCTGAGTTTACTACAAGATGAGTTACTGTTGAAGGATTCGACCAGGGAGTTGAATCAGTATGCCGTACTAAAGGAGCACCCAACTGATCTGTTGATAATGTCAGCAAAAAGCCGATGTCTTGGTCGTGGAAATGTTTTCCATAGCGCCCTGGTGGAATGCGTTCGCCTGCATCGCTTCATCCTGCACTGTGCCGTGGAGCTGGAGAACCTCTTCAGCCCGTACTGCCTGGTCAAGTCCTTACAGATCACTTTCCAGCTTTGCTTGTTGGTCTTTGTGGGAGTTTCGGGGACTCGAGAGGTCCTGCGGATTGTTAACCAACTTCAGTACCTGGGACTCACCCTCTTCGAGCTCCTCATGTTCACCTATTGCGGTGAGCTGCTGAGTAGGCATAGCATTCGATCTGGAGATGCCTTCTGGCGAGGCTCATGGTGGAAACACGCCCAAGCCGAATTCCAGCACACTGGCGGCCGTTACTAGTGGATCCGAGCTCGGTACCAAGCTTG
